# General practitioners’ continuation and acceptance of medication changes at sectorial transitions of geriatric patients - a qualitative interview study

**DOI:** 10.1186/s12875-018-0855-x

**Published:** 2018-10-12

**Authors:** Anja G Strehlau, Michael Due Larsen, Jens Søndergaard, Anna B Almarsdóttir, Jens-Ulrik Rosholm

**Affiliations:** 1 Geriatric Department, Research Unit, Odense University Hospital, Sdr. Boulevard 29, 5000 Odense C, Denmark; 2 Centre for Clinical Epidemiology, Odense University Hospital, and Research Unit of Clinical Epidemiology, Institute of Clinical Research, University of Southern Denmark, Odense, Denmark; 3 Department of Pharmacy, Social and Clinical Pharmacy, University of Copenhagen, Copenhagen, Denmark; 4 Institute of Public Health, Research Unit of General Practice, University of Southern Denmark, Odense, Denmark

**Keywords:** Mesh compliant, Transitional care, Patient discharge summaries, Follow-up care, Drug prescriptions, Geriatrics, General practitioners

## Abstract

**Background:**

Follow-up in general practice on medication initiated during hospitalisation is often perceived to be inadequate, which leads to unintended drug interaction and over- or underdosage of medication. Little is known about General Practitioners (GPs’) views on medication changes during the transition from hospital to primary care. We conducted a qualitative interview study to understand GPs’ views on the medication changes made for their patients by hospital physicians in a geriatric ward and the GPs’ actions after discharge.

**Methods:**

Qualitative semi-structured interviews comprising ten GPs from general practices in the Region of Southern Denmark, using a phenomenological approach. The GPs were selected strategically based on the principle of maximum variation. The analysis process was a cross-sectional analysis based on a phenomenological analysis.

**Results:**

The GPs identified many reasons for the lack of medication continuation, including miscommunication between hospital doctors and GPs and delayed discharge letters. Several factors were involved, including patients not taking responsibility for their medication, no structure for follow-up visits to their GPs and for the renewal of their prescriptions.

**Conclusion:**

The main reason for the poor continuity of medication changes for geriatric patients at sector transition was neither the GPs’ deliberate actions of removing the patients’ medications, nor the patients’ lack of compliance or of willingness to take the medication. It is largely due to procedural errors in the follow-up on the patient after discharge, due to the lack of a structured process and due to miscommunication between the primary sector and the hospital.

**Electronic supplementary material:**

The online version of this article (10.1186/s12875-018-0855-x) contains supplementary material, which is available to authorized users.

## Background

One of the cornerstones of modern geriatric care is a comprehensive geriatric assessment (CGA), which is a multidimensional diagnostic process. The likelihood of multiple overlapping problems necessitates an assessment across several domains and therefore involves several aspects – one of them being a medication review [1, 2]. CGA is carried out by geriatricians, and ideally, changes in medicine during hospital stays should be continued after discharge.

However, studies have documented a frequent and significant problem in the follow-ups in general practice on the medication changes made in hospitals [3–11]. A register study from Denmark showed that only about one third of the changes made in hospitals was followed up in general practice [3]. We learned from a prospective cohort study that, for only 3% of patients, discharge letters adhered to the national guidelines, and that the median time delay between the discharge date and the date of sending the discharge letters was 6 days [12].

This insufficient follow-up may cause drug interactions, over- and underdosage and thus inadequate treatment of patients [4, 13, 14]. There is still a lack of knowledge of the reasons for the insufficient follow-up, and a qualitative interview study can help to understand those reasons. Therefore, we aimed to describe firstly GPs’ views on the information on and handling of the medication changes made for their patients during hospitalisation and secondly GPs’ actions regarding medication changes for patients recently discharged from a geriatric ward in a hospital.

## Methods

We conducted a qualitative interview study comprising ten GPs in the Region of Southern Denmark. The interviews were conducted from may to September 2016. General practice is the corner stone of Danish primary health care. General practice is embedded in a universal tax-funded health care system, in which GPs and hospital services are free at the point of use [14].

The GPs were selected strategically based on the principle of maximum variation, comprising geographic location, gender, age, and affiliation to a solo or a partnership practice. Six GPs were female, and one was in a solo practice. Their location was spread over the Region of Southern Denmark. They varied from younger GPs having just completed their training as GPs to the more experienced GPs. The GPs were contacted and invited by letter stating the purpose and estimated duration of the interview along with the abstract of the above-mentioned register study [3]. The participants received a fee of 1,000 Danish Kroner (approximately 130 Euros).

### Interview procedure

The semi-structured interviews were conducted by one of the authors (AGS). The interview guide was partly based on the above-mentioned register study of Larsen et al. [3], clinical experience and a literature review. The interview guide was adjusted as new themes emerged. The main themes in the interview guide comprised the informants’ considerations in relation to the follow-up on patients after discharge and on medication changes initiated during hospitalisation. We also explored how the GPs handle prescriptions. (The interview guide can be found in the Additional file 1). The interviews were audio-recorded and transcribed. The duration of the interviews was about 35–40 min. All interviews were listened to by an independent individual, who simultaneously read the transcriptions to ensure consistency with the audio recordings. All interviews were treated as confidential.

### Analysis

The analysis process was a cross-sectional analysis based on Giorgi’s 4 steps of the phenomenological analysis [15]. All interviews were processed and labelled and meaning-bearing units were selected according to themes. Subsequently, a decontextualization was carried out, themes were divided into subgroups, and reflections on the meaning, message and relevance of each subgroup were made. The condensation comprises the opinions expressed by the informants. These were transformed into a shorter formulation. Long statements were abbreviated to few words containing the meaning of the statement.

A recontextualization of the condensed text was carried out, and summaries for each subgroup were prepared. Then quotes from the meaning-bearing units were deployed.Finally, summaries of new headlines describing contents and all interviews were read over again to challenge the results obtained and to find contradictions in the conclusions. Field notes were used in the process. All quotes were translated literally, and three dots were used to show if they had been shortened.

## Results

The results are divided into two main themes, namely *Theme 1: Information about hospitalisation and discharge, and the role of the discharge letter and Theme 2: Follow-up on medication changes initiated during hospitalisation.* Several sub-themes are described. All main themes are divided into subthemes. These are described individually under each main theme, each having their own points and content. During the interviews there were 5 main themes, as seen in the appended interview guide. These were processed and divided into subthemes as the results were analyzed.

### Sub-theme: The GP’s awareness of the discharge

There was no uniform structure for the procedure following the discharge of a patient from hospital. Usually, the GP received a discharge summary after a period varying greatly from days to weeks. The GP could also be contacted by a home nurse informing them that their common patient had been discharged, in case the nurse had questions about the changes in medication. Another possibility was that the patient’s family called the GP to inform him/her that their relative was hospitalised. Some of the GPs spontaneously contacted their patients after discharge. *“If it’s someone I have a close connection to, I might call them, but it’s not that I always call my patients just because someone has been hospitalised”* . Another way for GPs to be informed about a patient’s hospitalisation was if the patient attended an emergency medical centre and was admitted, after which the patient’s GP would receive a notice from the hospital. One GP stated that it might just be one sentence: *“The patient is too sick to stay at home - admission to geriatric unit required”* .

If the home nurse, who often has the initial contact with the elderly patient after discharge, called the GP before the discharge summary was received, the GP would have difficulties adjusting the medicine and planning for the patient. *“It would increase the quality of patient care if the GP received the discharge summary as soon as the patient had been discharged”*.

### Sub-theme: The discharge letter

The GPs all stated the importance of the hospital doctors making their key statements at the beginning of the discharge letter, e.g. marked as ‘For the attention of the GP’ or in the conclusion. *“I admit that, when reading the discharge summaries, I read the first three lines thoroughly. Then I skim the rest, and then I read the conclusion. It is easy for details to get lost because I read so many [discharge letters] every day”*.

Another obstacle was the organisation of reading the discharge letters sent to each practice, for example during a GP’s vacation when a colleague would have to read the GP’s patients’ discharge letters: *“Information may get lost because colleagues read discharge letters for my patients. There is room for errors here…”*.

The GPs were asked for solutions, and some expressed an interest in receiving a discharge summary with a certain kind of foreseeable structure. They wished that the key statements were at the beginning or in the conclusion, and that there was an explanation of what the scans and other examinations showed, and of why the medication was started or stopped. This would help the GPs inform the patients and thereby hopefully increase the continuation of medication initiated during hospital admissions.

### Sub-theme: Structure for follow-up after discharge

When the patient had been discharged from the geriatric unit at the hospital, there was substantial diversity in how and if a follow-up session with the patient’s GP was arranged. For most of the GPs, patients with chronic diseases visited them for a check-up once a year, when the GP would see if there were any changes made in the patients’ medication. A few GPs visited the patients in their homes after discharge. According to the GPs, the best way to ensure the follow-up was that the patients, family members or the home nurse called the GP to make an appointment for a visit to the clinic. One GP stated that he never spontaneously contacted patients. None of the GPs stated that a follow-up after admission to the hospital happened automatically, not even if the patient’s medication had been changed dramatically during the hospitalisation. Most GPs said that the network for follow-ups was quite ‘tight’ because relatives or home nurses were often involved when it came to elderly patients, and they made sure that these patients made appointments with the GP. The ones who were left out were elderly persons who lived alone and did not get any help from home nurses.

#### Theme 2: Follow-up on medication changes initiated during hospitalisation

##### Sub-theme: Miscommunication/good communication

The lack of structure after discharge caused frustration among some GPs. They described miscommunication as being the largest problem in the follow-up on patients after discharge. For example, if a new medication treatment was initiated during hospitalisation, the patients had been told in the hospital that the GP had to come to their homes for blood samples and follow-up. Some GPs were frustrated by such promises made to patients from the hospital – because it was not possible for them in any way to fulfil these promises after discharge. *“Some of the messages coming from those who write them at the hospital are characterised by the fact that they have not tried to work in general practice and do no not understand the workflow here”* .

The GPs felt that the follow-up on medication changes could be improved if it was stated very clearly in the discharge letter what should happen, e.g. that blood samples were needed after 2 weeks, or if they would get a call from their colleagues at the hospital concerning their common patient*.* Most GPs stated the importance of a thorough discharge summary with an explanation: *“It may very well be obvious for the geriatric doctor, but it is important for me to know so that I can explain it to the patient. It improves compliance”*. *“Polypharmacy is a challenge. Which medicine could be discontinued, and which should be discontinued?”*

During the follow-up, all GPs tried to involve their patients in the changes made in their medication during admission. For most GPs, it was important to explain each prescription thoroughly to the patients. Some GPs printed out lists of the medicine and explained it to the patients.

Some GPs believed that they would change the medication initiated by geriatric doctors during hospitalisation if they felt that this was in the best interest of the patient. Several GPs said that they would observe whether side-effects emerged, and the initiated medication had to be stopped. Only a few GPs were very reluctant to change the medicine initiated by geriatric doctors at the hospital.

##### Sub-theme: Patient responses to drug changes

The GPs had different views on how the elderly responded to changes in their medicine. Some were positive and often had confidence in authority figures. Most elderly were positive about changes and accepted them, if explained in a way they understood in a peaceful setting. The patients also had great respect for hospital doctors, even though they did not always understand why changes in their medication were made, and for how long they were supposed to follow these changes.

The elderly tended to prefer the medication they were used to. They did not like generic substitutes with different names, even though they often understood that it contained the same medication. This can compromise compliance significantly as exemplified by this GP’s statement: *“… I have had elderly people in my practice who took a double dose of the same medication because it had two different names, or they stopped taking it because they thought it was the wrong kind of medicine”* .

##### Sub-theme: Suggestions for improvement

To cope with the lack of a structured process, several GPs suggested a closer cooperation with geriatric doctors. Some suggested a form of hot line for GPs to a geriatric doctor, whom they could call and discuss their patients with. This collaboration could prevent hospital admissions of their patients or prevent re-admissions of patients who had just been discharged. Often, they were in doubt, and it would improve the quality of the patient treatment to be able to call a geriatric colleague. Three GPs wished it would be someone whom they knew and collaborated with on a regular basis so that they could function as a team. One GP points to geriatric doctors’ possibility of giving GPs information about current evidence and guidelines, for example about what to be aware of when treating elderly with non steroidal anti-inflammatory drug (NSAID) etc. Almost all GPs would prefer that the doctors at the geriatric units called them if they had a common patient needing close follow-up. They also regretted that their elderly patients often had to be admitted to emergency units, instead of going straight to geriatric units. One GP stressed that it would increase the possibilities of following-up on and treating the patient in the primary sector if the GPs themselves could order scans and other examinations and not always would have to refer a patient to the hospital for these services.

##### Sub-theme: Renewal of prescriptions

When a patient needed renewal of a prescription, they, relatives or the home nurses often called the GP by phone or ordered their prescriptions online. Subsequently, a member of the GP’s staff checked the journal to ensure that the prescription complied with the list of regular medication and prepared the prescription for approval by a GP before it was sent to the pharmacy. The approval procedure differed among GPs.

*“I approve the prescriptions by placing my elbow on the approve button - I hope you can hear the irony - but there is a certain amount of truth in it because our staff members are so meticulous”*.

It was a problem that the patient sometimes believed that, when a prescription expired, they should not take the medicine anymore. If the GPs had written a prescription with an expiration date, there was often no problem, unless the patient had forgotten to renew the prescription. If the GPs had written a prescription and marked the medication as permanent, the medication continued till someone acted and ended the prescription. The GPs very much disagreed on whether they should help the patient actively to remember to renew their prescriptions, or whether this was the patient’s own responsibility. As part of this solution, some GPs suggested teams of doctors with both geriatric and general backgrounds, who could pay home visits on a regular basis after discharge to make sure that prescriptions were correct, and that the patient knew which medicine to take and for how long.

## Discussion

Our prior understanding of the subject matter was that there was reason for concern that the follow-up in general practice after the patient’s discharge from hospital was insufficient, based on an epidemiological register-based study, which was undertaken in Denmark [3]. Furthermore, the literature on reasons for this discontinuity was sparse. A number of interventions have been tried to solve these problems. A literature review found that, among the most effective ones, were medication reconciliation; electronic tools to facilitate, quick, clear, and structured summary generation; discharge planning; shared involvement in follow-up by hospital and community care providers; use of electronic discharge notifications; and web-based access to discharge information for general practitioners. It is clear that the GPs in our study saw one of these effective options as a way forward in their endeavours to diminish the problems of patients having recently been discharged from hospital [16].

Overall, we found that there is a lack of a structured process after discharge, which results in discontinuity for patients going through sectorial transitions. This is worrying if it is evident that the changes in drug treatment made during admission should be continued in general practice. In the light of the many problems with geriatric patients’ drug use, it can also be argued that the drug treatment, which is based on a comprehensive geriatric assessment, should continue in general practice after discharge. A GP expressed the thought that the reason for the lack of follow-up on the medication plan made by the hospital was miscommunication including delayed discharge letters. Among the other reasons stated by the GP, there were patients not taking responsibility for their medication and problems when renewing their prescriptions.

The impact of sectorial transitions on medication changes has been examined in some studies. Various problems have been highlighted, one being the importance of good discharge letters from the hospital to the patients’ GPs. These must arrive immediately after discharge to enable awareness of the medication changes initiated during hospitalization [6, 8, 9, 17]. Most GPs in our study stated that they often did not receive the discharge letter in time. Consequently, the GP did not know which treatment had been initiated or discontinued during hospitalisation. The discharge letter is an essential platform for communication and provides information not only about changes in medication, but also about other important matters which may be lost.

Larsen et al. [3] showed that only one third of the medication changes initiated by the hospital was continued in general practice. Our study aligns with this finding from register-based studies and points out that there are multiple problems to be addressed regarding follow-up on patients after discharge. According to our informants, a structured process for follow-up on geriatric patients is surely needed. The GPs agree that home visits to patients after discharge are a way of ensuring more continuity of treatments initiated during hospitalisation. It can therefore be speculated that factors, such as a high workload and remuneration systems, may be reasons for the lack of home visits and follow-up.

The GPs in this study mention miscommunication between physicians working at the hospital and GPs as one of the important issues in the follow-up on patients after discharge. The frustrations among some GPs in our study about promises made to patients during hospitalisation which could not be fulfilled in general practice were noteworthy. The GPs in our study disagreed on how a follow-up visit at home should be initiated, and on how often they should visit patients at home. However, there was a broad consensus that visits were an effective way of ensuring the follow-up on a patient’s medications after discharge. A way of ensuring the quality of the follow-up and of reducing the above-mentioned frustration could be to align the information which patients receive at the hospital with a general agreement on how to follow up among GPs.

They proposed various solutions, of which the most prominent ones were communication with the geriatric doctors and to build teams working together to collaborate on patient care.

This study has some weaknesses and limitations. A bias was that the interviews have been carried out with a range of clinical working GPs who may already have reflected on their own actions in practice since they agreed to participate. A consideration was that the time limit in the interview may have led to GPs refraining from talking *too* much about the surveyed subjects, which reduces the amount of new issues which may arise during a qualitative study. There were ten GPs participating in this study, which is a small, but for this study, appropriate sample of GPs to represent this group of physicians. The selection of GPs in the study can be considered as a strength as it was strategically based on the principle of maximum variation, comprising variables such as geographic location, gender, age, and affiliation to solo or partnership practices. Finally, the participating doctors will have received patients and discharge letters from various hospital departments and not just from the geriatric department, which means that some of the statements may relate to a variety of departments and not just to the geriatric department.

The anticipated significance of this project for future perspectives is that it can open the way for examining ways to improve the follow-up after sectorial shifts for other patients than geriatric patients. The suggestions made by the GPs in this study are interesting. For example, working in teams with a geriatric doctor, having the opportunity for direct telephone contact with them, or paying home visits after discharge could all be possibilities worth studying to show the effect. These ideas can be used to design intervention studies which should be tested for effect using a mixed method approach.

## Conclusion

In conclusion, the main reason for the poor continuity of medication changes for geriatric patients in sector transition was neither the GPs’ deliberate actions to change the patients’ medications, nor the patients’ lack of compliance or of willingness to take medication. It is largely due to procedural errors in the follow-up on the patient after discharge resulting from the lack of a structured process and to miscommunication between the primary sector and the hospital staff.

## Additional file

Additional file 1: Interview guide. The main themes in the interview guide comprised the informants’ considerations in relation to the follow-up on patients after discharge and on medication changes initiated during hospitalisation. The interview guide was adjusted as new themes emerged. (DOCX 14 kb)

## Abbreviations

CGA: Comprehensive geriatric assessment
GP: General practitioner
NSAID: Non steroidal anti-inflammatory drug
