# Supplementary material for: General practitioners’ continuation and acceptance of medication changes at sectorial transitions of geriatric patients - a qualitative interview study
Source: BMC Fam Pract. 2018 Oct 12;19:168. doi: 10.1186/s12875-018-0855-x (PMC6182783; doi:10.1186/s12875-018-0855-x)
Supplement: Supplementary file 1 — Interview guide. The main themes in the interview guide comprised the informants’ considerations in relation to the follow-up on patients after discharge and on medication changes initiated during hospitalisation. The interview guide was adjusted as new themes emerged. (DOCX 14 kb) [file 12875_2018_855_MOESM1_ESM.docx]

Interview guide

Understanding GPs’ views on the challenges of sectoral shift to general practice and medication changes made for their patients by hospital physicians in a geriatric ward. GPs’ actions after discharge.

5 Themes:

Patient hospitalisation:

- What happens in your practice when a patient is discharged from a geriatric department?

- How are you informed about hospitalisation of your patients?

- Who initiates the first contact after a hospitalisation?

- How do you handle discharge letters?

Follow up on medication changes:

- Who initiates follow-up of any medication changes after hospitalisation?

- How do you asses patients compliance in relation to medication changes made in hospital?

- What is the patient's attitude toward drug changes after hospitalisation?

- How do you plan to follow-up on any medication changes made during hospitalisation?

- How is your patient involved in the follow-up on medication changes?

Prescriptions:

- How do you renew a prescription?

- Who monitors when the prescription is expiring?

- Which staff is involved in the renewal of a prescription?

- How du you monitor whether the prescription should be renewed?

- FMK:

- How do you use FMK? (Joint medication register)

- What is the significance of FMK for follow-up on medication adjustments?

- What is your opinion on FMK?

- Perspective:

- What can be done better from the hospital to optimize these drug changes?

- Suggestions for optimizing follow-up on medication changes and adjustments?
